# Health conditions and lifestyle risk factors of adults living in Puerto Rico: a cross-sectional study

**DOI:** 10.1186/s12889-018-5359-z

**Published:** 2018-04-12

**Authors:** Josiemer Mattei, Martha Tamez, Carlos F. Ríos-Bedoya, Rui S. Xiao, Katherine L. Tucker, José F. Rodríguez-Orengo

**Affiliations:** 1 Department of Nutrition, Harvard TH Chan School of Public Health, 665 Huntington Ave, Bldg. 2, Boston, MA 02115 USA; 2 Department of Internal Medicine, Hurley Medical Center, Flint, MI USA; 3 FDI Clinical Research, San Juan, PR USA; 4 Department of Biochemical and Nutritional Sciences, University of Massachusetts, Lowell, MA USA; 5 Department of Biochemistry, School of Medicine, Medical Sciences Campus, University of Puerto Rico, San Juan, PR USA

**Keywords:** Puerto Rico, Health disparities, Chronic disease, Lifestyle risk factors, Population health

## Abstract

**Background:**

Puerto Rico is experiencing an economic and healthcare crisis, yet there are scarce recent and comprehensive reports on the population’s health profile. We aimed to describe prevalent risk factors and health conditions of adults living in Puerto Rico and assess their interrelationship.

**Methods:**

Participants (*n* = 380) aged 30-75y recruited from a 2015 convenience sample in primary care clinics in the San Juan, Puerto Rico metropolitan area answered cross-sectional interviewer-administered questionnaires on sociodemographic characteristics, lifestyle behaviors, self-reported medically-diagnosed diseases, health services, and psychosocial factors. Anthropometric measures were obtained. Logistic regression models assessed factors associated with having ≥2 cardiometabolic conditions or ≥ 2 chronic diseases.

**Results:**

Most participants had completed ≥college education (57%), had household income <$10,000/y (60%), received government-assisted food benefits (51%), and had health insurance (93%). Nearly 20% reported smoking, 27% alcohol use, 74% light/sedentary physical activity, 51% sleeping difficulties, and 36% self-rated fair/poor diet. Social support was moderate, and 53% screened positive for depressive symptomatology. Abdominal obesity was observed in 33% of men and 76% of women (*p* < 0.0001). Self-reported medically-diagnosed conditions included hypertension (39%), anxiety (30%), obesity (28%), arthritis (26%), hypercholesterolemia (24%), depression (22%), respiratory problems (21%), and diabetes (21%). Higher odds of having ≥2 cardiometabolic conditions (37%) was observed among participants aged ≥50y, with sedentary physical activity, and self-rated fair/poor diet. Odds of having ≥2 chronic diseases (62%) were higher among ≥50y, sleeping difficulties, > 2 h/day television, and self-rated fair/poor diet. Participants obtained (79%) and trusted (92%) health information from physicians. While most participants with a cardiometabolic condition reported receiving medical recommendations on diet (> 73%) and physical activity (> 67%), fewer followed them (< 67% and < 53%, respectively), yet most adhered to medication treatments (> 73%). Participants following medical recommendations were more likely to report healthy vs. poor behaviors (90% vs. 75%, self-rated diet); (73% vs. 56%, physical activity).

**Conclusions:**

Adults living in Puerto Rico have multiple lifestyles risk factors and high prevalence of chronic diseases, namely cardiometabolic and psychological conditions. Comprehensive epidemiological studies are needed to identify contributors to chronic disease, including lifestyle behaviors. Concerted multi-level public health and clinical programs should be prioritized to help this population improve their health.

## Background

Multiple studies have documented that Puerto Ricans living in the mainland United States (U.S.) have poor health behaviors and chronic conditions, compared to other Hispanic/Latino backgrounds, as well to the general U.S. population. Unhealthy lifestyle behaviors include smoking, low physical activity, and poor diet quality [1–3]. Similarly, high prevalence of obesity, diabetes, hypertension, arthritis, cardiovascular diseases, and depression has been reported for this group [1, 2]. Conversely, Puerto Ricans on the U.S. mainland tend to have higher household income and higher rates of health insurance coverage, employment, and educational attainment than other Hispanics/Latinos, yet these remain lower than the general U.S. population [1, 4].

Despite studies reporting health-related data for Puerto Ricans on the U.S. mainland, less is known about the health behaviors and conditions presented by adults living on the island of Puerto Rico, a U.S. territory. The Behavioral Risk Factors Surveillance System (BRFSS) tracks some – but not comprehensive – statistics, and these may be limited by sampling methods and response bias [5]. Still, results indicate social and health disadvantages. For example, median household income is under $20,000, and unemployment is high, despite relatively high levels of educational attainment [6]. Self-reported consumption of fruit (44%) and vegetables (76%), and of meeting physical activity guidelines (8%) suggest unhealthy lifestyle behaviors [7]. In comparison, in the U.S. states, 48% of households earn >$50,000 annually, and self-reported consumption of fruit (60%) and vegetables (80%), and meeting physical activity guidelines (20%) are higher than in the island [7]. Disparities in common chronic conditions also exist, with prevalence of 42% in Puerto Rico vs. 31% in U.S. for hypertension; 39% vs. 36% for high cholesterol, 16% vs. 10% for diabetes; and 9% vs. 6% for coronary heart disease or myocardial infarction; additionally, 66% of island residents have self-reported body mass index (BMI) consistent with overweight or obesity [7]. High prevalence of cardiometabolic conditions and behavioral risk factors were shown in a 2005 probabilistic cross-sectional study of Puerto Ricans aged 21-79y that used questionnaires and physical and laboratory measures [8, 9].

Aside from the aforesaid study, there is a dearth of comprehensive epidemiological studies assessing risk factors and chronic conditions among adults in Puerto Rico. The island is experiencing an economic crisis and a shift in sociodemographic structure [10] which have intensified in the aftermath of hurricane María in 2017, making it crucial to report recent and valid data on multi-level contributors to chronic diseases that would depict the situation in the island before such events. Such information would help identify public health priorities and potential solutions, as well as help promote further research studies on the contributors to chronic diseases within the island’s context, especially before-after the natural disaster. Descriptive data are necessary to help set the appropriate indications and contra-indications for clinically-relevant action [11]. Thus, we aimed to describe the prevalence of sociodemographic, lifestyle, psychosocial, and healthcare risk factors, as well as prevalent chronic health conditions, along with their interrelationships, in a convenience sample of adult men and women living in Puerto Rico.

## Methods

### Study population, setting, and design

The Puerto Rico Assessment of Diet, Lifestyle, and Diseases (PRADLAD) study is a cross-sectional survey of a convenience sample of 380 adults living in Puerto Rico, conducted in 2015 with the goal of assessing lifestyle risk factors and health conditions among adults in the island. Study design and methodology have been described in detail previously [12]. Participants were patients waiting for a medical appointment, or visitors, recruited from three primary care clinics (a community clinic (*n* = 206); a research-based clinic (*n* = 101); and a city hospital clinic (*n* = 73), selected for their strategic locations, facilities, and wide patient representation) in the San Juan metropolitan area. Eligible individuals had to be living in Puerto Rico at the time of the study and for at least 10 months of the previous year, aged 30-75y, and able to answer questions without assistance. All participants provided written informed consent. The Institutional Review Board at Harvard T.H. Chan School of Public Health, Ponce Health Sciences University, University of Massachusetts Lowell, and Northeastern University, approved the study.

### Data collection

Questionnaires were administered by trained, Spanish-speaking interviewers in a private room in the clinic. Data were collected and managed using the secure, web-based electronic data capture tool ‘Research Electronic Data Capture’ (REDCap).

Questionnaires were based on instruments used by the Boston Puerto Rican Health Study [2] and the National Health and Nutrition Examination Survey [13]. General demographic and socioeconomic questions included household composition, educational attainment, marital status, work history, household income, food security and food assistance, and use of communications technology. Participants were asked whether a physician or health professional had ever diagnosed a list of chronic conditions. If so, we obtained information on medications, time of diagnosis, and current status of the disease. Participants with a diagnosis of any of five main cardiometabolic conditions (hypertension, diabetes, obesity, high cholesterol, or heart disease) were asked if they had received and/or followed medical advice on diet, physical activity, or medication use for each condition. Additional medical questions included menopausal status (for women), family history of main chronic diseases, health services, health insurance, and self-rated health status.

We assessed detailed information on history, frequency, amount, and type of smoking and alcohol use with questionnaires previously used in this population [2]. A physical activity score was calculated as the sum of hours spent on typical 24-h activities, captured using a modified Paffenbarger questionnaire, multiplied by weighing factors for each activity level. We asked for total hours of sleep over a 24-h period and difficulty falling asleep.

The 14-item Perceived Stress Scale was used to measure perception of life as stressful [14, 15]. We used the Center for Epidemiology Studies - Depression Scale to assess depressive symptomatology; high depressive symptomatology was defined as a score ≥ 16 [15, 16]. Perceived social support was assessed with the 12-item Interpersonal Support Evaluation List [17, 18], including three subscales: appraisal, belonging, and tangible support. Participants who reported a diagnosis of diabetes were asked the Diabetes Social Support Questionnaire-Family Version [19] to assess perceived family support for diabetes management.

Self-reported weight, height, and systolic and diastolic blood pressure were recorded. BMI was calculated by dividing self-reported weight by height squared. Waist and hip circumference measures were available for 316 participants using standardized protocols [20] in duplicate or thrice if there was more than 1 cm of difference between measurements. We used the average of the measurements as the final value. Abdominal obesity was defined according to U.S. guidelines (≥102 cm men, ≥88 cm women), with a second cutoff (≥94 cm men, ≥80 cm women) suggested by the International Diabetes Federation (IDF) for populations of European or Sub-Sahara African heritages [20]. We calculated waist-to-hip ratio by dividing the waist by hip measurement; a waist-to-hip ratio of ≥0.90 in men or ≥ 0.85 in women was deemed as high [20]. Weight status classifications by BMI were: underweight (15.0 to 18.4 kg/m^2^), recommended weight (18.5 to 24.9 kg/m^2^), overweight (25.0 to 29.9 kg/m^2^), or obesity (≥30 kg/m^2^) [20].

### Statistical analysis

Descriptive characteristics for all participants and by sex were assessed. Differences by sex were tested using chi-square for categorical variables or t-test for continuous variables. We created a combined variable for participants who reported ‘ever receiving’ medical advice on diet for any of the conditions probed, similarly for medical advice on physical activity or medication use. We also created a variable for ‘currently following’ medical advice on diet, physical activity, or medication use for any of the conditions combined. Differences by ‘ever receiving’ or ‘currently following’ medical recommendations for diet, physical activity, or medication use by self-rated diet quality, physical activity status, or actual medication use, respectively, were tested using chi-square.

Multivariable logistic regression models were used to determine sociodemographic and lifestyle factors correlating to the likelihood of having two or more cardiometabolic conditions or two or more chronic diseases, given that risk of mortality and quality of life increases for individuals with multiple prevalent conditions [21–23]. Cardiometabolic conditions were defined as the sum of current self-reported medically-diagnosed hypertension, obesity, high cholesterol, high triglycerides, pre-diabetes, diabetes, and heart disease or stroke. Chronic diseases were defined as the sum of current self-reported medically-diagnosed cardiometabolic conditions plus thyroid disease, arthritis, osteoporosis, anxiety, depression, cancer, bladder or kidney disease, gastrointestinal disease (including liver), eye-related diseases, sleep apnea, respiratory diseases, and physical disabilities. Definitions were based on WHO [24]. The reference categories were having none or one condition. Odds ratio (95% confidence interval) were obtained by categories of age, sex, marital status, educational attainment, household income, monthly food insufficiency, smoking status, drinking status, sleep hours, sleep difficulties, physical activity level, hours spent watching television, self-rated diet quality, and clinic site.

All analyses were done using SAS software version 9.4 (SAS Institute Inc.; Cary, NC). Significant differences were considered at a two-tailed *p* < 0.05.

## Results

Mean (SD) age was 51.5y (11.2), and 66% of the sample was female (Table 1). The sample was mostly comprised of self-identified Puerto Ricans, with a subgroup of Dominicans or people from the U.S. or other Latin American countries. More women than men were not married or living with a partner, and had higher educational attainment. There were no other significant differences by sex in other sociodemographic characteristics. Most participants reported a household income under $10,000, were retired or stay-at-home, and had health insurance. Nearly a quarter of the sample reported living alone. Frequent food insufficiency was reported by nearly 15% of the sample; more than half reported receiving benefits from the Supplemental Nutrition Assistance Program. While nearly all participants reported having a cell phone, fewer reported texting or using the internet. Most participants reported living on the island most for their lives, yet more than a quarter reported living in the mainland U.S. for at least one continuous year, and nearly 1 in 5 reported planning to move from the island permanently, mostly to the mainland U.S. (92%). Main reasons for planning to move were to improve quality of life (82.3%), to seek employment or for professional and financial reasons (72.6%), for personal reasons (69.4%), or to seek health services (54.8%).

**Table 1:** Sociodemographic characteristics of 380 adults 30–75 y/o living in Puerto Rico

Characteristic	All (*n* = 380)	Men (*n* = 131)	Women (*n* = 249)	*p*-value
Age, years	51.5 (11.2)	51.8 (11.3)	51.3 (11.2)	0.69
Rural area of residence, %	15.9	18.3	14.6	0.34
Ethnicity, %				
Puerto Rican	81.6	81.7	81.5	0.87
Dominican	14.5	13.7	14.9	
United States/Other	4.0	4.6	3.6	
Marital status, %				0.03
Married/living with partner	42.8	52.0	38.0	
Divorced/separated/widowed	20.9	15.8	23.6	
Single	36.3	32.3	38.4	
Education, %				
No schooling or < 8th grade	11.9	15.2	10.3	0.008
9th – 11th grade	6.2	7.2	5.7	
12th grade	24.9	33.6	20.5	
Some college or college degree	46.3	34.4	52.5	
Graduate school	10.6	9.6	11.1	
Household income, %				
$0–$10,000	59.9	52.8	63.7	0.18
$10,001–$20,000	21.2	26.9	18.1	
$20,001–$50,000	14.1	13.9	14.2	
> $50,000	4.8	6.5	3.9	
Employment, %				
Currently employed	36.6	36.6	36.6	0.63
Retired/stay-at-home	48.2	45.8	49.4	
Unemployed	15.3	17.6	14.1	
Health insurance, %				0.83
Government-assisted	55.4	53.3	56.6	
Private	37.1	38.3	36.4	
No health insurance	7.6	8.3	7.1	
Living alone, %	24.7	29.8	22.1	0.10
Number of people in household	2.6 (1.8)	2.5 (1.8)	2.6 (1.8)	0.50
Food security and assistance, %				
Frequent food insufficiency	14.5	15.5	14.0	0.93
SNAP food assistance^a^	51.1	44.4	54.5	0.07
WIC food assistance^a^	6.8	3.2	8.6	0.05
Use of technology, %				
Has cellphone	91.3	92.0	90.9	0.73
Uses texting	75.5	75.4	75.5	0.99
Uses Internet	55.2	53.6	56.0	0.66
Migration history, %				
Lived in PR most of their life	88.6	87.2	89.3	0.41
Lived in US at least one year	27.8	32.8	25.1	0.12
Plans to move from PR	17.6	20.7	16.0	0.28

Shown as mean (standard deviation) or percent, as assessed from a cross-sectional convenience sample of 380 adults aged 30-75y recruited in 2015 from primary care clinics in the San Juan, Puerto Rico metropolitan area

^a^Determined as positive if any member of the household currently received benefits from the Supplemental Nutrition Assistance Program (SNAP) or the Women, Infant and Children program (WIC)

Nearly two-thirds of participants had measurement-based abdominal obesity, as defined by current U.S. guidelines; the prevalence was 75.6% when using ethnic-specific IDF criteria, similar to the prevalence of high waist-to-hip ratio (Table 2). Self-reported data showed a lower percent of BMI-based overweight and obesity, relative to central obesity. More women than men were significantly classified with abdominal obesity or BMI-based obesity. Women were also more likely than men to be non-smokers and to not drink alcohol. Other lifestyle factors were similar for men and women. Overall, the prevalence of unhealthy lifestyles included sedentary habits, short (< 7 h) or long (> 8 h) sleeping hours, sleep difficulties, and current smoking. Only 25% of participants had a yearly flu shot. Health and diet quality were self-rated as fair or poor by 40.1% and 35.6% of participants, respectively. Finally, the main sources of health information included physicians, TV/radio, health professionals, and newspapers/magazines. Physicians and health professionals were highly trusted. Women were more likely to seek and trust health information on the internet than men. Nearly 53% of participants screened positive to depressive symptomatology. Scores for perceived stress, social support, and diabetes emotional support were moderate; with the social support subscale for ‘appraisal’ (i.e.: receiving advice or guidance) having the highest mean score. There were no differences by sex in any of the psychosocial measures.

**Table 2:** Lifestyle and health-related risk factors and psychosocial measures of 380 adults 30–75 y/o in Puerto Rico

	All	Men	Women	*p*-value
Abdominal obesity U.S. cutoff, %^a^	61.3	33.0	76.2	< 0.0001
Abdominal obesity IDF cutoff, %^a^	75.6	55.1	86.4	< 0.0001
High waist-to-hip ratio, %^a^	76.8	77.6	76.4	0.81
BMI, %^b^				
Underweight	11.1	14.6	9.2	< 0.0001
Recommended weight	46.7	63.4	38.1	
Overweight	21.8	14.6	25.5	
Obesity	20.4	7.3	27.2	
Physical activity, %^c^				
Sedentary	43.5	40.0	42.2	0.10
Light	30.7	26.7	32.7	
Moderate/Vigorous	25.9	33.3	22.1	
Habitual relaxation exercises, %	7.8	9.8	6.8	0.47
Sleep, hours/day	7.0 (1.7)	6.9 (1.6)	7.0 (1.7)	0.51
Less than 7 h/day	37.7	39.4	36.8	0.20
7–8 h/day	50.7	53.3	49.4	
More than 8 h/day	11.6	7.4	13.9	
Sleep difficulties, %				
Always	22.0	26.2	20.0	0.31
Occasionally	28.7	25.4	30.5	
Rarely	49.3	48.4	49.8	
TV watching, hours/day	3.7 (2.7)	3.8 (3.0)	3.7 (2.6)	0.76
Time seated, hours/day	4.3 (3.2)	4.5 (3.8)	4.1 (2.9)	0.34
Smoking status, %				
Never smoker	66.4	54.8	72.4	0.002
Former smoker	15.2	22.2	11.5	
Current smoker	18.4	23.0	16.1	
Alcohol consumption, %				
Non-drinker	50.8	37.8	57.6	< 0.0001
Former drinker	22.4	35.4	15.6	
Current drinker	26.8	26.8	26.8	
Yearly influenza vaccination, %	25.7	25.6	25.7	0.59
Self-rated health, %				
Excellent/Very good	24.5	26.9	23.3	0.40
Good	35.4	37.7	34.1	
Fair/Poor	40.1	35.4	42.6	
Self-rated diet quality, %				
Excellent/Very good	30.6	28.2	31.9	0.31
Good	33.8	35.1	33.1	
Fair/Poor	35.6	36.6	35.1	
Source of health information, %				
Physician	79.2	80.9	78.3	0.55
Health professional	62.0	63.1	61.5	0.77
Newspapers/magazines	60.7	56.5	62.9	0.22
TV/Radio	63.7	59.5	65.9	0.22
Internet	55.2	46.6	59.7	0.01
Friends/family	51.7	50.4	52.4	0.71
Trust this source, %				
Physician	91.7	93.1	91.0	0.48
Health professional	77.9	79.4	77.1	0.62
Newspapers/magazines	52.7	52.7	52.6	0.99
TV/Radio	47.5	45.0	48.8	0.49
Internet	46.7	39.2	50.6	0.04
Friends/family	46.0	44.5	46.7	0.69
Perceived stress score^d^	21.7 (7.7)	21.5 (8.0)	21.8 (7.5)	0.71
Depressive symptoms score^d^	17.6 (12.6)	16.4 (11.9)	18.3 (12.9)	0.19
% with depressive symptoms^e^	52.6	51.3	53.2	0.74
Social support score^d^	24.7 (7.1)	24.0 (7.6)	25.0 (6.8)	0.19
Appraisal	8.4 (2.8)	8.2 (2.9)	8.5 (2.7)	0.22
Belonging	8.2 (2.8)	8.1 (2.9)	8.2 (2.7)	0.64
Tangible	8.0 (2.6)	7.8 (2.7)	8.2 (2.5)	0.19
Diabetes emotional support score^f^	14.1 (7.5)	14.5 (7.7)	13.9 (7.4)	0.71

Shown as mean (standard deviation) or percent, as assessed from a cross-sectional convenience sample of 380 adults aged 30-75y recruited in 2015 from primary care clinics in the San Juan, Puerto Rico metropolitan area

^a^*n* = 316; Abdominal obesity defined as waist circumference ≥ 102 cm men or ≥ 88 cm women according to U.S. guidelines, or ≥ 94 cm men or ≥ 80 cm women according to International Diabetes Federation (IDF) criteria. High waist-to-hip ratio defined as > 0.90 men; > 0.85 women

^b^Classified from self-reported weight and height as underweight (15.0–18.4 kg/m^2^), recommended weight (18.5–24.9 kg/m^2^), overweight (25.0–29.9 kg/m^2^), or obesity (≥30.0 kg/m^2^)

^c^Sedentary physical activity defined as a score < 30, light activity as 30 to < 40, and moderate/vigorous activity as ≥40, as captured using a modified Paffenbarger questionnaire

^d^For all scores, higher values of the score are indicative of higher psychosocial marker. Possible ranges are 0–56 for perceives stress score, 0–60 for depressive symptoms (measured with Center for Epidemiology Studies Depression Scale), 0–36 for social support (measured with12-item Interpersonal Support Evaluation List-12)

^e^Depressive symptomatology defined as a score ≥ 16 in the Center for Epidemiology Studies Depression Scale

^f^Diabetes Social Support Questionnaire-Family asked only to those who reported diabetes diagnosis (*n* = 78). Possible range is 0–25; higher score indicates higher diabetes support

The main medically-diagnosed chronic conditions reported by participants were hypertension, anxiety, obesity, arthritis, hypercholesterolemia, depression, respiratory problems, and diabetes, all of which were reported by at least 20% of participants (Table 3). Women had significantly higher prevalence of obesity, arthritis, hypercholesterolemia, thyroid diseases, and osteoporosis, but lower prevalence of hepatitis than men. The majority of those diagnosed with hypertension, diabetes, or thyroid diseases used medication for the condition; medication use was lower for the other conditions. The majority of participants who reported ever being diagnosed with a condition reported still having the condition at the time of the study, except for hypertension and cancer. Family history of hypertension, diabetes, and heart disease were commonly reported; there were no significant differences by sex.

**Table 3:** Percent of self-reported medically-diagnosed conditions and family history for 380 adults 30–75 y/o in Puerto Rico

	Ever diagnosed by a physician				If ever-diagnosed, currently uses medication^a^	If ever-diagnosed, currently has the disease^a^
Self-reported medical diagnosis	All	Men	Women	*P*-value	All	All
Hypertension	39.2	38.6	39.6	0.85	92.4	36.9
Anxiety	29.7	26.2	31.5	0.29	54.1	88.7
Obesity	27.7	14.7	34.8	< 0.0001	6.9	90.1
Arthritis	25.6	18.6	29.2	0.03	46.7	95.5
Hypercholesterolemia	23.8	16.1	27.8	0.01	64.4	89.4
Depression	22.1	21.6	22.3	0.88	61.7	82.3
Respiratory problems	20.9	15.9	23.5	0.09	56.0	89.6
Diabetes	20.7	24.2	19.0	0.23	91.0	94.9
Thyroid diseases	17.7	9.7	21.8	0.004	84.6	90.8
Gastrointestinal diseases	17.2	12.0	19.8	0.06	71.4	85.3
Pre-diabetes	15.2	15.1	15.2	0.97	55.4	88.5
Hypertriglyceridemia	14.7	15.7	14.2	0.70	44.2	94.3
Eye diseases	12.7	14.9	11.6	0.37	22.2	89.0
Physical impairment	12.7	16.8	10.6	0.09	66.0	93.5
Hepatitis	12.4	23.4	6.6	< 0.0001	42.2	88.1
Sleep apnea	11.3	15.1	9.3	0.10	47.5	92.3
Heart disease	10.0	11.5	9.2	0.49	60.0	76.3
Cancer	4.0	1.6	5.4	0.09	31.3	13.3
Fatty liver disease	7.5	6.7	8.0	0.68	25.9	88.5
Osteoporosis	5.2	1.6	7.1	0.02	42.1	94.7
Family History						
Hypertension	73.3	70.6	74.7	0.40	–	–
Hypercholesterolemia	41.9	39.7	43.0	0.54	–	–
Diabetes	63.9	63.1	64.4	0.80	–	–
Heart diseases	52.9	48.1	55.4	0.17	–	–

Shown as percent, as assessed from a cross-sectional convenience sample of 380 adults aged 30-75y recruited in 2015 from primary care clinics in the San Juan, Puerto Rico metropolitan area

^a^Calculated for those participants who responded ‘yes’ to have been ever diagnosed with the disease

Two or more self-reported current cardiometabolic conditions were noted in 37% of the sample; 25% had one cardiometabolic condition and 38% had none. For chronic diseases, 62% had two or more, 17% had one, and 21% had none. Logistic regression models were used to determine sociodemographic and lifestyle factors associated with having two or more cardiometabolic conditions or chronic diseases (Table 4). Higher odds ratio (95% CI) of having ≥2 cardiometabolic conditions (vs. > 2) were observed among participants age ≥ 50y vs. age < 50y), with sedentary physical activity (vs. light/moderate activity), and self-rated fair/poor diet (vs. excellent/very good). Participants with ≥2 chronic diseases (vs. > 2) were more likely to be aged ≥50y, watch television > 2 h/day (vs. ≤2 h/day), had sleeping difficulties (vs. rarely), and self-rated fair/poor diet. Sensitivity analysis excluding sleep apnea or physical disabilities showed identical results. No other sociodemographic or lifestyle factors were observed to be significantly associated with multiple conditions.

**Table 4:** Likelihood of having two or more conditions by risk factors among 380 adults 30–75 y/o in Puerto Rico

	≥2 Cardiometabolic conditions^a^	≥2 Chronic diseases^a^
Age ≥ 50y (vs. <50y)	2.63 (1.55, 4.46)	3.43 (1.99, 5.90)
Female (vs. male)	1.03 (0.60, 1.76)	1.13 (0.65, 1.99)
Single or divorced/widowed (vs. married)	1.37 (0.83, 2.27)	1.06 (0.62, 1.80)
High school or lower education (vs. ≥ college)	1.21 (0.69, 2.10)	1.11 (0.62, 1.98)
≤$10,000 household income (vs. > 10,000)	1.02 (0.54, 1.94)	1.00 (0.51, 1.98)
Monthly food insufficiency (vs. never)	1.12 (0.68, 1.86)	1.18 (0.69, 2.02)
Current smoker (vs. never or former)	0.77 (0.40, 1.51)	1.09 (0.54, 2.18)
Current drinker (vs. never or former)	1.27 (0.71, 2.25)	1.56 (0.84, 2.89)
< 7 or > 8 h sleep/night (vs. 7–8 h)	1.12 (0.67, 1.85)	1.21 (0.71, 2.05)
Sleep difficulties always/occasionally (vs. rarely)	1.20 (0.72, 2.10)	2.88 (1.67, 4.98)
Sedentary physical activity (vs. light or moderate/vigorous)	1.91 (1.12, 3.25)	1.44 (0.81, 2.57)
> 2 h/day TV watching (vs. ≤2 h/day)	1.30 (0.74, 2.28)	1.80 (1.01, 3.21)
Good/fair/poor self-rated diet quality (vs. excellent/very good)	2.01 (1.19, 3.39)	2.34 (1.37, 4.02)

^a^Shown as odds ratio (95% confidence interval) obtained from a multivariable logistic regression model adjusted for the variables shown, as assessed from a cross-sectional convenience sample of 380 adults aged 30-75y recruited in 2015 from primary care clinics in the San Juan, Puerto Rico metropolitan area. Two or more cardiometabolic conditions (*n* = 139) was defined as the sum of current self-reported medically-diagnosed hypertension, obesity, high cholesterol, high triglycerides, pre-diabetes, diabetes, and heart disease or stroke. Two or more chronic diseases (*n* = 234) was defined as the sum of current self-reported medically-diagnosed cardiometabolic conditions plus thyroid disease, arthritis, osteoporosis, anxiety, depression, cancer, bladder or kidney disease, gastrointestinal disease (including liver), eye-related diseases, sleep apnea, respiratory diseases, and physical disabilities. The reference categories were having none or one condition/disease

**Fig. 1:**
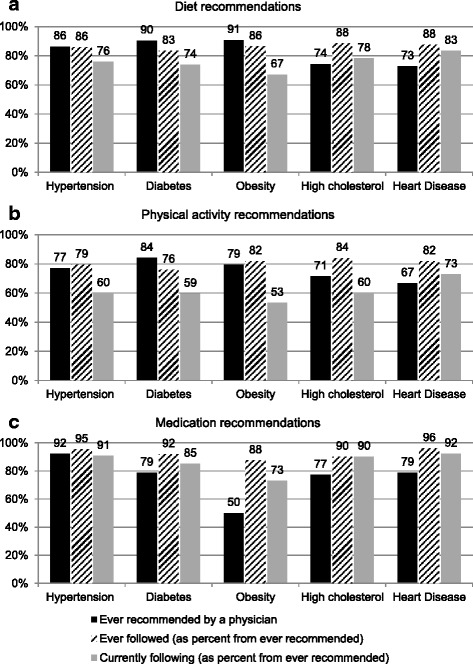
Percent of adults in Puerto Rico who reported receiving and following medical recommendations as treatment for cardiometabolic conditions. Panel ‘**a**’ shows recommendations for diet, panel ‘**b**’ for physical activity, and panel ‘**c**’ for use of medication. Black bars represent the percent of participants reporting if the corresponding recommendation was ever given by a physician; slanted bars represent the percent of participants who ever followed the recommendation (as a percent of those who ever received the recommendation); dotted bars represent the percent of participants who were currently following the recommendation (as a percent of those who ever received the recommendation). Shown as percent for a subsample of 139 adults who responded having at least one cardiometabolic condition, from a cross-sectional convenience sample of 380 adults aged 30-75y recruited in 2015 from primary care clinics in the San Juan, Puerto Rico metropolitan area

Diet and physical activity recommendations, as given by their physician, were most frequently reported by participants with hypertension, diabetes, and obesity, while use of medication was most frequently noted by participants with hypertension (Fig. 1). While most participants had received advice on diet and physical activity for each condition, fewer had followed or were currently following this advice, especially for diabetes, obesity, and hypertension; yet most adhered to medication treatments. Self-rated excellent/very good diet quality (vs. poor/fair) was more often reported among participants who were currently following medical recommendations on diet for any of the five chronic conditions probed (90% vs. 75%, *p* = 0.013); similar results were observed for light/moderately physical activity vs. sedentary (73% vs. 56%, *p* = 0.029) among participants currently following medical recommendations on physical activity, and for actual medication use (96% vs. 73%, *p* < 0.0001) among currently following medication recommendations. Ever receiving medical recommendations was more often reported among participants using medications (98% vs. 54%, *p* < 0.0001), but there was no difference by self-rated diet quality (*p* = 0.89) or physical activity status (*p* = 0.87).

## Discussion

Participants from a cross-sectional study of adults, aged 30-75y from the San Juan, Puerto Rico metropolitan area, had poor socioeconomic and lifestyle factors as well as high prevalence of multiple chronic conditions, with differences by sex in several characteristics. Paradoxically, most adults had attained some college education or higher, yet reported low annual household income. Low income may have been observed because most participants were retired or stay-at-home, with an additional 15% unemployed. More than half of the sample received food assistance and most had government-assisted health insurance. Our observations agree with recent statistics from the island [6], and relate to the current economic crisis that has stalled employment and wages [10]. The education and income disparity was notable for women, who had significantly higher education than men, yet tended to report lower household incomes despite reporting the same employment rate.

The most striking differences by sex were observed for anthropometric measures; more than twice the percent of women (76%) than men (33%) had abdominal obesity. Our results are comparable for men, but higher for women as observed in a probabilistic cross-sectional study from 2005 (51% for women, 37% for men) [8]. We observed higher prevalence of abdominal obesity for men when using IDF-based criteria (55%), which use a lower waist circumference cutoff based on differential fat distribution of people with European and Sub-Saharan African heritages. Waist-to-hip ratio was similarly high for both sexes. Both anthropometric measures denote elevated accumulation of abdominal fat, which is a strong predictor of multiple chronic diseases particularly diabetes [25], and thus merits urgent attention in this population.

When obesity was classified based on self-reported weight and height, women also showed higher prevalence of overweight (26%) and obesity (27%) compared to men (15 and 8%, respectively). Self-reported medically-diagnosed presence of obesity tended to agree with these numbers. However, a study that used measurement-based BMI reported higher prevalence of overweight and obesity among both women (33% and 44%, respectively), and men aged 21–79 years (40% and 38%) [9]. Self-reported 2014 BRFSS data showed similar results as the aforesaid study except for women with obesity, which was lower (30%) [7]. This suggests that participants in our study may have underreported their weight. Notably, men in our study were more likely to be underweight or have normal weight, and the only condition that was reportedly higher in men than women was hepatitis, for which treatment could lead to weight loss [26].

The majority of participants self-rated their health as fair/poor, and we observed high prevalence of sedentary behaviors and tobacco use, and low vaccination for influenza. These observed frequencies are similar to those reported in BRFFS except for current smoking, which was higher in our study (11% vs. 18%) [7]. From among all U.S. states and territories, Puerto Rico had the lowest percent of people reporting good/excellent health and of adults 65y or older receiving a flu shot, and the highest percent of adults reporting no leisure-time physical activity in the BRFSS [27]. Additionally, nearly half of participants reported short or long sleep time and some sleeping difficulties, and the majority rated their diet as fair or poor, suggesting that lifestyle and health-related behaviors tend to be poor in this sample.

Psychosocial questionnaires suggest that adults in Puerto Rico have moderate perceived stress and social support, as well as emotional support for those with diabetes. Similar scores using the same scales have been reported for perceived stress among Puerto Rican middle-aged and older adults living in Boston, MA [15], and for social support for Puerto Rican adults in the U.S. [18]. However, more than half of the sample presented with depressive symptomatology. Using the same scale, Puerto Rican men in Boston had a similar mean depression score as in our study, but women in Boston had higher mean score than women in our study (22 vs. 18); the results were significantly different by sex in the Boston study [15]. Puerto Ricans in the U.S. were observed to have the highest percent of depressive symptomatology (38%; lower than observed in our study) among Hispanics/Latinos; these higher odds of having high depressive symptoms persisted after adjusting for demographic, lifestyle, and co-morbid conditions [28].

The self-reported prevalence of the assessed clinical diagnoses in our study were generally similar to those reported by BRFSS [6, 7]. Only 40% of individuals with diagnosed hypertension reported currently having it; medication use was high for these individuals and our data suggest that they tend to adhere to it. It is possible that their blood pressure has been regulated by medication and they perceive their hypertension to have resolved. The high prevalence of diagnosed diabetes in the island agrees with previous reports [7, 29, 30]. Puerto Rico has the highest percentage of people with diabetes among all U.S. states and territories [27]. Notably, an additional 13.2% of adults in Puerto Rico have been estimated to have undiagnosed diabetes as detected by laboratory measurements [30], indicating that diabetes screening, prevention, and control must be prioritized in the island. Family history of hypertension and diabetes were frequently reported. We have previously shown that Puerto Rican adults carry risk alleles in higher frequency and protective alleles in lower frequency than non-Hispanic whites, as assessed from variants involved in major metabolic and disease-relevant pathways [31].

We identified several lifestyle behavioral contributors to multiple cardiometabolic conditions and multiple chronic diseases, including poor sleep, sedentary behaviors, and poor self-rated diet. However, no sociodemographic factors were significantly correlated. While the limited sample size, or reverse causality, may be a factor in the inability to detect significant social determinants, the results suggest that unhealthy lifestyle behaviors may play a larger role in shaping chronic conditions in this population. A study among women from San Juan, PR showed that physical activity was associated with lower odds of metabolic syndrome, but not social determinants such as marital status [32], and in a cross-sectional study of adults in San Juan, PR, lower educational status, no alcohol intake, and low physical activity were associated with metabolic syndrome, but these associations attenuated after controlling for biomarkers [33].

Despite the collapsing health care system in Puerto Rico that has left the island with low availability and quality of services [34, 35], 76% of adults still seek yearly checkups and 70% have a personal health care provider to manage their health [7]. Our study shows that participants sought – and trusted – health information from a physician or health professional. This was also denoted by the generally high percentage of participants with a cardiometabolic condition that reported ever or currently following treatment recommendations given by their physician. While the recommendations were mostly followed for medication use, adherence to diet and physical activity advice was lower. Notably, ‘currently following’ but not ‘ever receiving’ medical advice for diet and physical activity was more likely noted among those reporting doing such healthy behaviors in the questionnaires (excellent self-rated diet or light/moderate physical activity), suggesting that delivering medical advice may not be sufficient for patients to adopt healthy behaviors and continued guidance, as well as other tangible or motivational support, may be needed. Bidirectional relationships may also be operating, as those with poorer healthy habits may be more likely to receive medical advice to improve behaviors [36]. Still, these observations provide an important opportunity for primary and secondary prevention of chronic conditions through health care providers. Adapted lifestyle interventions that have proven more effective for diabetes prevention than medication have been successfully implemented among Latinos in the U.S. in both clinical and community settings [37, 38]. Other sources of health information included media, internet, and advice from family or friends; however trust in these sources was lower. Use and trust on the internet was particularly low in men, which agrees with previous reports [7, 39].

In general, the poor lifestyle behaviors and high prevalence of chronic disease persist for Puerto Ricans on the island as well as the U.S. mainland. However, direct comparisons between the groups show marked differences in some factors, such as higher health care coverage and educational attainment but lower income in the island [29]. Previous studies have reported lower prevalence of diabetes, smoking, influenza vaccination [29], and incidence of cancer [40] in the island, as well as substantial variability in causes of death [41]. Additionally, Puerto Ricans living in the U.S. but born on the island have been reported to have similar rates of mood and anxiety disorders but higher overall mortality rates than their U.S.-born counterparts [42]. Importantly, the distinctive pattern of circular migration for Puerto Ricans needs to be taken into account as it may be related to social, economic, behavioral, and health-related dynamics [43]. Our study showed that 28% of participants had lived on the mainland U.S. for at least one year and nearly 1 in 5 planned to move away permanently, mainly seeking better jobs, quality of life, and health services.

The cross-sectional design of this study serves to depict participants’ characteristics as of 2015, yet it limits inferences on causality. The convenience sampling in primary clinics from the San Juan metropolitan area reduces the generalizability of our results, and it is possible that those seeking primary care services were either more health-conscious or needed clinical care due to pre-existing conditions. However, health insurance coverage in Puerto Rico is high, thus most people had access to care in the recruitment clinics, and their diverse locations improved the sociodemographic representation of our sample [12]. The prevalence of chronic conditions reported in our study were similar to those reported by BRFSS and previous studies, suggesting that we captured accurate occurrence of disease among adults. Using assessment instruments that were previously validated in this population also improved the accuracy of data. Nonetheless, generalizability of results should be considered cautiously.

## Conclusions

We illustrate the current social, lifestyle, and health conditions of adults in a convenience sample of adults attending three clinic locations in San Juan, in Puerto Rico, which will be instrumental in identifying priorities for public health programs and policies to help this population reduce substantial health needs. Priority should be given to improving socioeconomic status, promoting healthy lifestyle behaviors, and addressing cardiometabolic conditions namely hypertension, obesity, and diabetes, as well as mental health. The identified contributing factors to some of these conditions suggest a larger influence from unhealthy lifestyle behaviors than from social determinants. Concerted multi-factorial efforts across the socio-ecological model are needed to address the health disparities present in the island – from interventions at the individual level to community- and population-based programs. Engaging policy-makers and government officials will be instrumental, as the multiple socioeconomic disadvantages and high dependence on government-assisted services may impact behaviors and health outcomes. Health care officials should also be on board, as individuals seek, trust, and follow their health recommendations. Our study provides timely and recent data to inform preventive efforts that may positively impact the health status of Puerto Rico residents, which is relevant and crucial given the financial and health care crisis affecting the island.

Our study opens the door to multiple lines of public health research, including the need to assess additional health risk factors in Puerto Rico, analyze the association of the observed risk factors and health outcomes within the context of the island, and expand assessment to the rest of the island, especially as geographical variance in prevalence of diabetes has been shown [44]. Importantly, our study builds on the evidence that the profile of health behaviors and outcomes of Puerto Ricans may differ between their place of origin and the mainland U.S. Public health officials and researchers must take these nuances into consideration to better target interventions and programs that account for the specific context and needs of this population.

## Abbreviations

BMI: Body mass index
BRFSS: Behavioral risk factors surveillance system
IDF: International Diabetes Federation
REDCap: Research electronic data capturing
US: United States
